# Circulating tumor cells: overcoming challenges of detecting a needle in a haystack

**DOI:** 10.37349/etat.2025.1002321

**Published:** 2025-05-29

**Authors:** Zhuldyz Myrkhiyeva, Kuanysh Seitkamal, Zhannat Ashikbayeva, Assiya Taizhanova, Daniele Tosi, Aliya Bekmurzayeva

**Affiliations:** University of Campania “Luigi Vanvitelli”, Italy; ^1^Laboratory of Biosensors and Bioinstruments, Center for Life Sciences, National Laboratory Astana, Nazarbayev University, Astana 010000, Kazakhstan; ^2^School of Sciences and Humanities, Nazarbayev University, Astana 010000, Kazakhstan; ^3^School of Engineering and Digital Sciences, Nazarbayev University, Astana 010000, Kazakhstan

**Keywords:** Circulating tumor cells, cancer, liquid biopsy, detection, biosensor, commercial assays

## Abstract

Circulating tumor cells (CTCs) are cancer cells that are detached from the primary and metastatic tumor site and invade the bloodstream. Most importantly, CTCs are the key players in the development of metastasis. As one of the main components of liquid biopsy, they may significantly contribute to improvements in early cancer diagnosis, monitoring response to therapy, and predicting recurrence of the disease. Although identifying and analyzing CTCs offers the potential for a real-time liquid biopsy, their detection is associated with a number of challenges, which mainly stem from three sources: complexity of the CTCs, complexity of the media (blood), and performance of the detection assays. Particularly, low concentration of the CTCs and the presence of a vast population of hematopoietic cells in the blood make their detection technically complex. The heterogeneity of the target cells and not enough sensitivity of the measuring platforms are also among major technical challenges in CTC detection. Therefore, this review aims to give an update on various methods developed for CTC isolation, including chip-based assays and biosensors. The work will elucidate various challenges associated with the isolation and detection of CTCs and showcase the studies that aimed to tackle them. A number of available commercial platforms for CTC detection and hurdles associated with their widespread applications in clinical settings will also be discussed.

## Introduction

Metastasis is the biggest threat to cancer patients, accounting for approximately 90% of all cancer fatalities. The blood of cancer patients holds the potential metastatic seeds—the source of metastasis [1]. These are cells detached from primary and metastatic tumor sites that enter the circulation via circulatory or lymphatic systems and spread to secondary organs, causing metastasis. Such cells were coined as circulating tumor cells (CTCs) [1]. The CTCs were first identified in 1869 by Ashworth, who observed cells in the blood of a metastatic cancer patient resembling primary tumor cells [2]. The discovery of CTCs as a multipurpose, minimally invasive biomarker has emerged as one of the most exciting advancements in contemporary cancer therapy [3]. CTCs are considered one of the main components of liquid biopsy, together with circulating nucleic acid molecules (DNA, RNA, microRNA), exosomes, and other tumor cells (tumor platelets and tumor endothelial cells) [4]. CTCs can become a helpful add-on target in cancer diagnosis and monitoring. Studies on CTCs have shown their potential significance, enabling the advancement of cancer care and optimization of therapeutic strategies. Extensive research in this area in recent years has demonstrated that CTCs could serve as a marker for predicting disease progression and survival in metastatic patients and potentially even in those with early-stage cancer [5–9]. Unlike invasive tissue biopsies, detection of CTCs offers the benefits of simple sample collection and the capability for real-time monitoring. In comparison to circulating tumor DNA (ctDNA), CTCs are intact tumor cells containing comprehensive omics data, including genomic, transcriptomic, proteomic, and metabolomic information [10]. Moreover, even in the absence of visible metastases, CTCs can provide insights into drug resistance mechanisms, guide therapeutic management [11], and signal the necessity or effectiveness of therapy. In clinical trials, they may serve as a surrogate endpoint marker [5], but they may also be targeted for treatment [11]. Research into novel methods for early detection of metastatic disease includes identifying CTCs, which offer a more reliable and less invasive approach compared to current techniques such as clinical exams, radiography, and serum tumor markers [12].

As a result, in the recent decade, significant efforts have been made to develop various methods to capture and analyze CTCs, and a number of reviews have been written on this topic in the last decade. Some of the papers [13] need an update with the accumulation of new studies in this area, while other works focus on a particular type of sensors, such as quartz crystal microbalance [14], electrochemical [15, 16], optical [17, 18] or chip-based platforms [19, 20]. Another group of works focuses on specific ligands used for CTC detection, such as aptamers [21] or the role of CTCs in a specific type of cancer [22].

**Figure 1:**
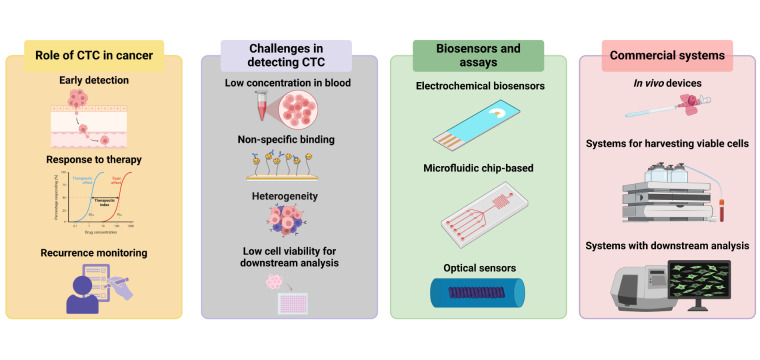
**An overview of the topics covered by the current work.** Created in BioRender. Bekmurzayeva, A. (2025) https://BioRender.com/k27i019. CTC: circulating tumor cell

This review aims at giving an update on the challenges associated with detecting CTCs and on the current biosensors and assays for their detection and characterization. It takes a different approach in addressing the topic of CTCs by focusing on the challenges and showcasing the studies that tackled them. This work also discusses commercially available platforms for CTCs and possible future perspectives, hoping to provide researchers and clinicians with informative insights into the usefulness of CTC detection in cancer management. An overview of the topics covered by this review is shown in Figure 1. Table 1 shows the main problems and brief explanation for which arise during CTC detection and provides some insights on how each of the issues can potentially be resolved.

**Table 1:** Challenges associated with the detection of the CTCs

**Challenge**	**Explanation**	**What requires**	**Ref.**
Rarity	As few as 1 CTC per billion blood cells Fewer than 1 CTC per 10 mL in early-stage cancer	Efficient enrichment Depletion of unwanted cells High detection accuracy High sensitivity Processing a large amount of blood	[23–26]
Heterogeneity	Phenotypic and genotypic variation across tumor types Marker-dependence (EpCAM) CTC size differs across cancer types; cell line-based filters may overestimate CTC size. Can miss CTCs with other characteristics, resulting in false negative results	Multiple biomarkers Combining more than one parameter: size- and label-based approaches Deformability-based microfluidics Combining with other components of liquid biopsy (cfDNA, exosomes) Label-free detection Real-time detection	[27–32]
Complex blood environment	Non-specific binding from blood cells and plasma proteins creates high background noise.	Antifouling surfaces Improving surface-cell interaction Dual-selective ligands	[33–35]
Low viability of captured CTCs	Delay in processing affects downstream analysis. High shear stress during isolation causes apoptosis/necrosis, damages CTCs, and reduces recovery.	Real-time detection Gentle capture via microfluidics or flexible biosensors Integrated culture systems Low-shear microfluidic designs to mimic physiological conditions	[33, 36–38]
Clinical implementation	Rapid processing needed Short tube storage times, logistical hurdles for analysis Freezing and thawing may cause CTC loss, aggregation, and reduced integrity. High cost of devices Performance of devices is limited.	Use preservative tubes Automate capture and analysis Miniaturized real-time biosensors Standardized cryopreservation Use of cryoprotectants Integrated biobanking protocols	[1, 37, 39, 40]

CTC: circulating tumor cell; cfDNA: cell-free DNA; EpCAM: epithelial cell adhesion molecule

### The role of CTCs in cancer: a brief overview

Currently, various cancer management approaches are under investigation, with particular emphasis on the role of CTCs in cancer progression. CTCs have several properties that contribute to their role in cancer metastasis. They often undergo epithelial-to-mesenchymal transition (EMT), where epithelial cells lose adhesion properties and gain mesenchymal traits, thus enhancing their motility and invasiveness [41]. CTCs also show stem-like characteristics like self-renewal and tumor-initiating abilities [42]. This allows them to form metastatic colonies and resist anti-cancer therapy [43]. Additionally, a few of CTCs interact with components of the blood microenvironment, including platelets, neutrophils, macrophages, and myeloid-derived suppressor cells, which help them evade immune detection, resist shear stress, and enhance metastatic potential [44]. Once CTCs shed from primary tumors into the bloodstream, they face numerous challenges that compromise their survival. The vast majority are short-lived and rapidly eliminated. However, a rare subset of cells manages to persist, possessing the capacity to initiate metastasis [45]. The key stages of CTCs include intravasation into the bloodstream, extravasation to bone marrow or other organs, dissemination throughout the body, and forming metastasis [33].

An observational study by Ried et al. [46] involving 600 CTCs tests on 542 patients showed that these cells were detected in 100% of cancer patients (*n* = 277) and 50% of asymptomatic patients with risk factors (132 out of 265), with follow-up scans identifying early cancerous lesions in 20% of screened patients and early prostate cancer in 50% of males with normal prostate specific antigen (PSA) levels but positive CTCs results, highlighting the value of CTCs analysis for early detection and monitoring. Numerous studies have demonstrated the high efficacy of detecting CTCs in diagnosis of breast [6], colorectal [7], and prostate cancer [5].

## Assays and biosensors

### Enrichment and detection using chip-based systems

The study of CTCs is a complex analytical process. In 1 mL of whole blood, the number of CTCs is usually less than 10 compared to 10 million white blood cells and 5 billion red blood cells [19]. To study these unique cells, complex enrichment methods are required to obtain their molecular and functional characteristics. Optimization of CTC enrichment methods implies a thorough analysis of various parameters, such as recovery efficiency (defined as the percentage of target cells successfully isolated), enrichment purity (the ratio of CTCs to contaminating cells), cellular viability maintenance, throughput capacity, pre-analytical requirements, operational costs, and analytical reproducibility. When choosing an optimal enrichment strategy, all these parameters should be taken into account as much as possible and correlated with the requirements of the subsequent research: molecular analysis, functional studies, or clinical diagnostics.

Currently, there are two main approaches to enrichment: biological methods, which are based on the biological properties of cells, and mechanical (biophysical) methods, which are based on physical principles. Biological enrichment strategies aim to exploit differences in the expression of antigens on the cell surface, such as epithelial cell adhesion molecule (EpCAM) and CD45. They also take into account metabolic activity and the ability to invade. In contrast, biophysical approaches rely on various physical characteristics of CTCs, such as their size (usually in the range of 12 to 25 micrometers in diameter, compared with 8–12 micrometers for leukocytes) [1], density, electrical properties (membrane capacity and cytoplasmic conductivity), and mechanical deformability.

The cells that are part of the tumor and circulate in the bloodstream have unique structural features. In particular, they have an increased ratio of a nucleus to the rest of the cell volume, which is a result of an increase in the size of the nucleus and a change in its shape [47]. The stiffness of the cytoskeleton also changes. These changes in the structure of cells contribute to their better penetration into tissues, allow them to survive under shear stress, and maintain the integrity of the membrane. They also affect the surface charge of the cell [48]. Due to these features, it is possible to develop methods for isolating CTCs.

A number of techniques have been developed to leverage the distinct physical properties of tumor cells compared to blood cells, enabling the enrichment and separation of CTCs from blood samples. Chip-based detection systems, for instance, offer a powerful and integrated approach to isolate and analyze these rare cells. These systems typically rely on microfluidic technologies that combine size-based filtration and/or antigen-specific capture, and sometimes even have on-chip culture capabilities [49]. Chip-based platforms can enhance sensitivity and specificity in CTC detection through precise fluid control and innovative designs, such as adding microstructures or functionalized surfaces, while minimizing sample loss [38].

Some of the chip-based platforms had integrated biosensors. Burinaru et al. [50] developed a microfluidic biosensor platform for the detection of CTCs from blood samples, based on a PDMS-encapsulated electrochemical impedance spectroscopy (EIS) system. The platform consists of an integrated microfluidic chip designed for label-free detection of CTCs by capturing impedance variations resulting from cell adhesion and interaction with the electrode surface. The microfluidic system features a functionalized area with interdigitated gold electrodes (GEs) coated with antibodies specific to EpCAM and anti-CD36 antibodies, enabling selective capture of CTCs while minimizing non-specific interactions. The biosensor utilizes an alternating current (AC) signal to measure impedance changes, allowing for the differentiation of CTCs from normal blood cells based on their distinct electrical properties. The device demonstrated high sensitivity, successfully detecting as few as three MCF-7 cells on the electrode surface. These results highlight the potential of the microfluidic device for clinical applications in cancer diagnosis and monitoring [50]. A method that uses magnetic nanoparticles to help capture and detect CTC is one of the other manufactured devices for removing uncommon cells from the bloodstream [23].

Hybrid magnetic and deformability-based microfluidic system for detecting CTCs was proposed by Chen et al. [51]. By targeting their larger size, reduced deformability, and EpCAM expression, the system combines physical filtration with magnetic techniques to isolate CTCs. Under a magnetic field, magnetic immunobeads enhance the capture efficiency of CTCs. Featuring gradually narrowing elliptical gaps, the microfluidic chip allows smaller, more flexible cells to pass through while retaining CTCs. With a high capture efficiency of over 90% at a flow rate of 3 mL/h and a cell viability of 96%, this hybrid design provides an efficient and reliable method for isolating CTCs from various cancer types, including gastric, colorectal, breast, and lung cancers [51].

### Electrochemical biosensors for CTC

Electrochemical biosensors are complex analytical devices that use the principles of bioelectrochemistry to detect and quantify certain biological interactions. The principle of operation of such systems is based on tracking changes in electrochemical processes that occur at the interface between the functionalized surface of the electrode, on which the bio-recognition elements are located, and the analyte [52, 53]. The design is based on the transducer element, which is usually an electrode modified with highly specific bio-recognition molecules such as antibodies, peptides, or aptamers. These molecules serve as a sensitive interface and provide interaction with the analyte. As a result of interaction with the analyte, changes occur in the electrical properties of the electrode-solution interface, which can be measured and interpreted as a result of biomolecular recognition. Depending on the measured electrical parameter, several types of detection mechanisms can be distinguished:

1. Potentiometric sensing. In this case, the accumulation of charge on the surface of the electrode is measured, which leads to a change in the electric potential in the absence of current.
2. Amperometric detection. This method allows you to determine the amount of current that is generated as a result of redox reactions at a fixed applied potential.
3. Impedance analysis. This method evaluates changes in the impedance of the electrode interface, usually using EIS.
4. Conductometric measurements. This method monitors changes in the conductivity of a solution caused by changes in ionic mobility or concentration.

The conversion system typically consists of a high-precision electrical generator that generates the signal and a sophisticated detection system that measures the corresponding electrochemical response. This configuration allows the detection of biomolecular interactions in real time without the use of tags. At the same time, the system has high sensitivity and specificity.

Table 2 shows some examples of electrochemical biosensors developed to detect CTCs. As can be seen from Table 2, most of the studies in the field of electrochemical sensors have only focused on MCF-7 cells as a CTC model, which demonstrates their importance for cancer diagnosis. Meantime, aptamers were frequently employed as receptors due to their high specificity and sensitivity. Although sample types ranged from PBS to human blood and clinical samples, for most of the sensors, measurements were not performed directly from clinical blood samples. Furthermore, several studies integrated microfluidic chips to enhance detection accuracy and throughput, and the detection limits varied from 0.43 cells/mL to 40 cells/mL depending on the technique and ligand used. Interestingly, most researchers detected EpCAM, but as demonstrated by numerous studies, EpCAM is not a universal marker for CTC detection. Gu et al. [54] used a light-addressable potentiometric sensor (LAPS) to detect CTCs in prostate cancer directly from whole blood. The system incorporated carboxylated graphene oxide (CGO) as a surface modification to enhance detection capabilities. The CGO-modified LAPS surface was functionalized with anti-EpCAM antibodies for specific CTC capture.

**Table 2:** Electrochemical biosensors developed for the detection of CTC

**Sensor type**	**Ligand**	**Cell/media**	**LOD**	**Main strengths**	**Ref.**
Impedimetric	Anti-EpCAM and CD36	MCF-7 cells in PBS	3 cells^※^	Integrated with a microfluidic chip Tested on canine mammary carcinoma	[50]
Amperimetric	EpCAM and MUC1 aptamers	MCF-7 cells in PBS	3 cells/mL	Dual-recognition-control for accuracy and sensitivity	[55]
Amperimetric	IDA aptamer	A549 cells	14 cells/mL	Novel aptamer was used. Integrated with a microfluidic chip Detection in clinical samples	[56]
Impedimetric	Anti-EpCAM	MCF-7 cells in PBS	NA	Electrically triggered release of viable cells	[57]
Impedimetric	Multifunctional peptide	MCF-7 cells	17 cells/mL	Linear response in 25% human blood	[58]
Impedimetric	Folate	HeLa cells in PBS and blood	0.43 cell/mL 52.24 cell/mL	Capable of differentiating patients of cervical and liver cancers	[59]
Electrochemiluminescence	Aptamers	MEAR cells	40 cells/mL	Selective, rapid detection Excellent reusability	[60]
CV/electrochemiluminescence	Aptamers	A549 cells	3 cells/mL	Highly efficient capture of cells Tested in whole blood	[61]
Nanopore-based	MUC1 aptamer	MCF-7 cells	1.25 cells/mL	Tested on clinical samples High recovery rate & accuracy	[62]

^※^ On sensor surface; CTC: circulating tumor cell; LOD: limit of detection; EpCAM: epithelial cell adhesion molecule; NA: not applicable

### Optical biosensors for CTC

Optical biosensors are a category of biosensors that utilize the interaction between an optical field and a biorecognition element [63]. The development of various optical biosensing platforms for the detection of CTCs was examined for detection of various cancers (Table 3). A comprehensive summary of various optical bio- and nano-aptasensors is provided in the work of Safarpour et al. [17]. Moreover, various commercially available devices employ fluorescence labels to detect CTCs. Mizutani et al. [40] developed a sensitive and specific fluorescence resonance energy transfer (FRET) biosensor to detect BCR-ABL kinase activity in live cells, enabling the identification of cancerous and drug-resistant cells and the evaluation of kinase inhibitor efficacy. For optical sensors, the detection of large analytes such as CTCs poses an additional challenge due to the size of the analyte under investigation, since cells have a size larger than the wavelength. Traditional label-free biosensors operate under the assumption that the functionalization layer, the bioreceptor, and the analyte binding on the surface of the device have altogether a size much smaller than both the wavelength and the skin depth of the medium [64]. Since cells have a size larger than the wavelength of operation, the biosensing analysis must take into account the light reflected and scattered by the cell into its surrounding environment [65]. For this reason, the detection of cells so far has been limited to sensors having a large surface area, such as tilted fiber Bragg gratings [66] or ball resonators [67]; by having a large surface for interaction, these sensors can integrate the response of the sensor over a large number of cells. However, this method requires the detection of a wide number of cells per unit of volume, and is impractical for the detection at low resolution and with fewer cells.

**Table 3:** Optical biosensors developed for the detection of CTC

**Sensor type**	**Ligand**	**Cell/media**	**LOD**	**Main strengths**	**Ref.**
Plasmonic TFBG	Anti-GPR30 antibodies	BT549	5 cells/mL	Simple structure Easy to manufacture Small size	[25]
Optical liquid crystals	EpCAM aptamers	MDA-MB-231 cells	5 cells/mL	Detection in human serum and whole blood	[68]
Smartphone-assisted biosensor using 3D polyurethane-GO	MUC1 aptamer	MCF-7 and HT29 cells	221 cells/mL	Portable and user-friendly Cost-effective Rapid detection	[69]

CTC: circulating tumor cell; LOD: limit of detection; EpCAM: epithelial cell adhesion molecule

## Overcoming challenges in detecting CTC

### Challenges associated with CTC isolation and detection

Current methodologies of CTCs detection face several challenges; two of the main challenges being the extremely low number of CTCs in blood samples and the absence of specific markers selectively identifying CTCs [70]. CTCs are often present in very small numbers—approximately 1 cell per 10^5^ to 10^7^ mononuclear cells—making their detection highly sensitive to technical limitations [71]. This means that just one CTC is potentially surrounded by about 1 million white blood cells and 1 billion red blood cells in each milliliter of blood [33]. Although isolation and enrichment processes could potentially alleviate this limitation, captured CTCs are often fragile and may be damaged during the process, which affects their viability and subsequent analysis [33].

Another major limitation is the heterogeneity of CTCs, exhibiting a wide range of genetic, molecular, and phenotypic variations depending on the tumor type and its stage. This heterogeneity complicates the detection process, as the markers used to isolate CTCs may not be present on all tumor cells [72]. Advanced tools like Bayesian classifiers can accurately group CTCs, but their complexity makes detection and analysis challenging [28]. Detection methods, like CellSearch™, rely on specific markers, such as EpCAM, which excludes EpCAM-negative CTCs. This marker dependence limits the detection of diverse CTC populations [29]. Additional issues include inefficiencies in capture, variations in size and phenotype, and non-specific labeling [34].

Shear stress presents another significant technical challenge in the detection of CTCs. Regmi et al. [36] demonstrated that modifying the microfluidic environment to generate varying levels of haemodynamic shear significantly impacted CTCs. High shear stress caused cellular damage, resulting in necrosis within the first 4 hours and apoptosis within 16–24 hours of circulation. This not only disrupted CTCs’ attachment but also damaged other epithelial-based cancer cells, including drug-resistant variants. Moreover, techniques like immunomagnetic separation require expensive reagents and specialized laboratory equipment.

Implementing CTC detection in clinical practice faces significant challenges, including the rarity and heterogeneity of CTCs, which complicate reliable isolation and characterization [39]. Detecting CTCs in early-stage disease is challenging because of their low concentration. In non-metastatic breast cancer (MBC), fewer than 1 CTC per 10 mL of blood is common, with five or more CTCs occurring in only 1–5.9% of cases. At diagnosis, CTCs are found in 20–25% of patients with localized disease when using a threshold of more than 1 CTC per 7.5 mL of blood, whereas metastatic cases require more than 5 CTCs per 7.5 mL for detection [73].

Cryopreservation is essential for enabling retrospective studies, batch processing, and long-term storage of CTC samples, ensuring their availability for future analysis. However, it poses challenges such as potential CTC loss during freezing and subsequent warming, cell aggregation due to DNA release, and the lack of standardized protocols to maintain sample integrity [37]. Additionally, the limited storage time of blood collection tubes necessitates rapid processing, creating logistical difficulties for transportation and delaying downstream analysis [39].

CTCs size varies across cancer types, with breast (12.4 µm) and prostate cancer CTCs generally larger (10.3 µm) than leukocytes (9.4 µm), while colorectal and bladder cancer CTCs tend to be smaller, 7.5 and 8.6 µm, respectively [27]. Mendelaar et al. [27] found that lab-grown breast cancer cells, at 18.4 µm in size, are significantly larger than patient-derived CTCs, which average 12.4 µm. This size difference can lead to inefficiencies in isolation techniques that are based on overestimated CTC dimensions.

### Innovative designs

One of the ways that chip-based platforms can improve the detection of CTCs is through fluid control and innovative design. Zhou et al. [38] demonstrated this approach by designing a microfluidic chip that integrates size-based isolation, capture, and on-chip culture into a single platform. The system uses a cell-separation channel to isolate larger CTCs from smaller blood cells via size-dependent migration, followed by a trapping chamber with diamond-shaped microposts to retain CTCs based on their size and deformability. This compact and efficient design streamlines the workflow by eliminating manual handling steps, reducing cell loss, and maintaining a high separation efficiency of over 94% for cells larger than 15 µm. With a cell viability of 97.4% during on-chip culture and support for functional assays like proliferation and migration studies for over 10 days, the platform demonstrates significant potential for liquid biopsy and personalized medicine applications [38].

“CTC-Chip” technology represents a microfluidic platform lined with EpCAM antibodies that utilizes unprocessed peripheral blood. It was specifically created to establish controlled conditions (laminar flow through microposts) in which cells would come into contact with antibodies that capture them [74]. By employing fluorescently tagged EpCAM and HER2 antibodies and incorporating optical fibers into the microfluidic apparatus, Pedrol et al. [75] developed a device for detecting breast cancer in blood.

### Improving sensitivity

Improving the sensitivity of the platforms detecting CTCs is one of the ways to tackle the challenge of rareness, some of which are sensor-specific. Thus, Peng et al. [55] coupled electrochemical sensors with rolling amplification reaction and DNA nanostructures to improve the sensitivity of the biosensor. They proposed a dual-recognition-controlled electrochemical biosensor for the accurate and sensitive detection of CTCs. In their design, the simultaneous presence of two tumor-associated cell-surface proteins, mucin 1 (MUC1) and EpCAM, triggers a dimer-like rolling circle amplification (RCA) process. This amplification generates long DNA products, which are captured by thiolated DNA strands (TDSs) immobilized on the electrode surface, resulting in a significantly amplified electrochemical signal and enabling highly sensitive quantification of specific CTCs. The proposed biosensor design offers three key advantages. First, the dual-recognition strategy significantly enhances the detection accuracy of specific CTCs and provides a promising approach for the precise prediction of tumor origin via liquid biopsy. Second, the method eliminates the need for special signal probes or materials, simplifying the analytical procedure and reducing experimental costs. Third, the integration of electrochemical techniques with RCA and TDSs greatly enhances sensitivity, enabling the detection of target CTCs at ultralow concentrations. The combination of RCA and TDSs achieves excellent sensitivity, allowing the detection of CTCs at concentrations as low as 3 cells per mL, demonstrating the biosensor’s potential for analyzing rare cancer cells.

A method for detecting CTCs was developed by Han et al. [76] using a flexible graphene-based biosensor fabricated on a polyethylene terephthalate (PET) substrate with graphene and silver paste electrodes. This electrochemical system detected ovarian cancer CTCs by measuring changes in electrical response when they interacted with the graphene surface. The biosensor demonstrated high sensitivity, detecting as few as 30 CTCs per milliliter, and provided rapid results within seconds. Its low cost, ease of fabrication, and ability to differentiate between ovarian cancer CTC concentrations made it a promising tool for early cancer diagnosis and monitoring.

For some of the CTC biosensors, sensitivity was enhanced using nanomaterials. Wan et al. [77] developed a graphene-based biosensor for detecting CTCs using a SiO_2_/Si substrate with graphene and GEs. This biosensor was based on measuring changes in electrical resistance when CTCs interact with the graphene surface and demonstrated high sensitivity, detecting as few as 1 to 10 CTCs, with response sensitivity increasing from 2% to 37% as the cell count rose from a few to 10,000.

Another way to increase the sensitivity is via using signal-enhancing probes. Thus, Pang et al. [35] developed a system that employs Fe_3_O_4_@Ag magnetic nanoparticles functionalized with anti-ASGPR antibodies to selectively capture CTCs and Au@Ag nanorods labeled with anti-GPC3 antibodies as surface-enhanced Raman scattering (SERS) tags for signal enhancement magnetically assisted SERS biosensor for detecting CTCs associated with hepatocellular carcinoma. The dual-selectivity provided by the antibodies ensures high specificity, while the dual-enhancement from the magnetic and plasmonic nanoparticles amplifies the Raman signals significantly. The biosensor demonstrated a detection limit as low as 1 CTC in 1 mL of blood and provided a linear detection range from 1 to 100 CTCs [35].

### Improving surface functionality

For example, Cetin et al. [78] developed a chip-based microfluidic system for detecting CTCs using self-assembled monolayers (SAMs) on gold surfaces functionalized with EpCAM antibodies. These antibodies selectively bind to EpCAM-positive CTCs through antigen-antibody interactions. Compared to alkanethiols without aromatic ring in the structure, alkanethiols with aromatic ring (4-aminothiophenol) were found to be a better option for improving cell capture due to better intramolecular interaction. The microfluidic channels facilitate controlled fluid flow, allowing efficient interaction between the functionalized surface and blood samples. This system also enhances precision, reduces sample volume requirements, and provides a robust platform for isolating and studying rare CTC populations.

Han et al. [58] developed an innovative two-step surface modification technique to create an antifouling electrochemical biosensor capable of detecting MCF-7 cancer cells directly in human blood. The approach is based on a multifunctional peptide that includes various domains for surface anchoring, conductivity modulation, antifouling, and specific recognition. The peptide organizes itself on the GE by attaching the N-terminal cysteine through a thiol bond, creating a strong Au-S bond. The peptide contains important functional components: a hydrophilic antifouling component that creates a protective hydration layer, reducing the nonspecific binding of proteins. The hydrophobic component was responsible for regulating conductivity. A specific recognition domain that provides selective interaction with MCF-7 cancer cells. A layer of poly(3,4-ethylenedioxythiophene) (PEDOT) was applied to the surface of this peptide. Thanks to the negatively charged amino acid, which is part of the peptide, it was possible to create a homogeneous conductive layer. This layer additionally prevents the nonspecific interaction of blood components.

Another approach included the use of specially synthesized hydrogel nanoparticles, which were synthesized using three components, each having its own function [79]. Zwitterionic sulfobetaine methacrylate served for reducing non-specific binding of unwanted cells, while methacrylic acid was used to provide a carboxyl group for further modification of the nanoparticles with the EPCAM-antibodies through carbodiimide chemistry. The third component was *N*,*N*'-methylene bisacrylamide, which provided nanostructures enhancing the interaction of the target cells with the surface. The nanoparticles were successfully tested using real clinical samples, detecting CTCs.

The next work of the same authors used another component as the third constituent for magnetic nanoparticle synthesis [*N*,*N*-bis(acryloyl)cystamine] [80] for a gentle recovery of captured cells via glutathione responsiveness. In another study, bioorthogonal microbubbles were used as an innovative substrate to capture CTCs using a nanorough antifouling surface [81]. This substrate was modified using bioorthogonal click chemistry to produce efficient enrichment and subsequent release of the cells.

### Label-free, real-time detection

Tumor dynamics in real-time can be monitored by analyzing the changes in CTCs (type and number) in the blood of patients to evaluate the treatment efficacy and to help find a personalized therapy approach [82]. Detecting real-time phenotypes of CTCs might be one of the ways in guiding cancer therapy, offering minimal invasiveness and convenient accessibility. Thus, HER2-targeted therapy in HER2-positive patients is not always effective in MBC therapy, mostly due to cancer heterogeneity, its constantly evolving nature, and subjectivity in evaluating the results of immunohistochemistry (IHC) and/or fluorescent in situ hybridization (FISH). Additionally, these standard diagnostic tools used to evaluate HER2 status are invasive (IHC) and hard to perform dynamically (both IHC and FISH). According to one study, both HER2 status and the treatment outcome of the patients were different when evaluated by standard method (IHC) and CellSearch system [83].

Chen et al. [25] proposed a plasmonic fiber Bragg grating biosensor for the detection of breast cancer cells, which, after coating with a gold nanofilm and immobilizing an antibody against GPR30, demonstrated the ability to detect breast cancer cells at concentrations as low as 5 cells/mL within 20 minutes, with a linear range of 5–1,000 cells/mL suitable for real-world CTC detection. Loyez et al. [66] developed an all-fiber plasmonic aptasensor for the detection of MBC cells, achieving real-time, label-free detection with a limit of detection of 49 cells/mL within 5 minutes, which was enhanced by functionalized gold nanoparticles.

SPR-based sensors are considered an attractive tool for CTCs detection, demonstrating great sensitivity, as shown in the work of Thawany et al. [84], where a gold-coated D-shaped optical fiber biosensor with immobilized anti-EpCAM antibodies successfully detected human liver cancer cells (Hep2G) in the range of 10–150 cells/mL. Needle-like cytosensor was developed for real-time, label-free detection of CTCs directly from the bloodstream based on functionalized needle [85]. Weng et al. [85] functionalized a stainless steel needle with EpCAM antibodies to capture CTCs while preserving their genetic integrity. This system used cyclic voltammetry and generated an electrochemical signal upon CTC binding. The biosensor showed high sensitivity, detecting 21 CTCs/mL.

## Commercially available platforms for CTC detection

Currently available commercial systems for CTCs can be divided into the following: 1) platforms for capturing CTCs (including those used for further separate downstream analysis); 2) systems for capturing and further downstream analysis of CTCs. Some of the examples of these systems, which were tested on the real clinical samples, will be discussed in this section.

### CellSearch™ system

CellSearch™ is based on EpCAM antibodies, which are designed to sample CTC in 7.5 mL of blood. For some time, it was the only test approved by the US Federal Drug Administration (FDA) for CTC quantification [29]. It was possible to classify MBC patients into risk groups using CellSearch™ [6]. This technology has set the bar high when it comes to CTC detection and remains the gold standard method [29]. Presence of five or more CTCs in the blood of patients diagnosed with MBC before initiating any therapy was associated with short overall and progress-free survival. Moreover, presence of the cells in 3–4 weeks after therapy initiation and during restaging is also correlated with prognosis [86]. However, images obtained from CellSearch™ can be interpreted subjectively, and no further analysis of cells is possible [87].

The number of studies using this system is very high, while controversies in the obtained results still remain; this prompted the scientific community to write systematic reviews and meta-analyses on this topic. Thus, a meta-analysis revealed a positive correlation between CTCs detected by the said technology with the prognosis of patients with esophageal carcinoma [88]. In case of gastric cancer, detected cells had a significant prognostic value and might be useful for prediction of poor responders to chemotherapy [89], while more recent study also showed the association with overall survival and disease-free survival/recurrence-free survival and progression-free survival in patients with the same type of cancer [90].

Over the years, this technology was combined with other methods in order to improve CTC detection: with immunomagnetic cell selection system (AdnaTest^®^) for diagnosis of metastatic colorectal cancer [91], with highly sensitive and specific qPCR-based assay for detection of mRNA levels of a gene related to an aggressive form of prostate cancer [92] and many other.

### CellCollector™ platform

Some of the commercial platforms are intended to be used in vivo. One of the early reports on using metal wires for capturing CTC from blood is a work by Saucedo-Zeni et al. [93]. A novel method for the in vivo isolation of CTCs from the peripheral blood of cancer patients using a functionalized and structured medical wire. Gilupi GMBH (www.gilupi.de) created one of the first devices for CTC isolation that addresses the challenge of processing enormous volumes of blood. In order to capture CTC. Their CellCollector™ is based on a medical guidewire composed of stainless steel coated with hydrogel, gold, and EpCAM antibodies. Furthermore, Gilupi™ provides customized CellCollector™ functionalized with an antibody of interest. A guidewire is a medical instrument frequently utilized to aid in the insertion of catheters, stents, and various interventional devices for both diagnostic and therapeutic reasons. The capturing method makes it possible to analyze collected cells further downstream using techniques including ex vivo cell culture, FISH, immunofluorescence (IF) analysis, and mutation analysis. After being captured, cells were treated with fluorescently labeled anti-EpCAM antibodies and CD45 antibodies (for ruling out leukocytes) and also exposed to various post-capture treatments such as immunostaining and observing under a fluorescent microscope [94], or chip-based digital PCR, DNA fluorescent-in-situ-hybridization [95].

When compared to CellSearch™, a method approved by the FDA for CTC detection, CellCollector™ has demonstrated better performance [96]. In certain studies that used the Gilupi guidewire, more CTCs were discovered in later stages of the disease, and a decrease in CTC count was noted following treatment (surgery) [94]. The Gilupi device may therefore be helpful for tracking the effectiveness of treatment. Post-capture techniques may be useful for identifying treatment targets and researching resistance mechanisms. It is argued that cancer patients typically undergo painful and time-consuming procedures (MRI, CT, biopsies), which may result in infection or bleeding, even though simply drawing blood is easier than placing a guidewire for 30 minutes [97].

### Parsortix^®^ system

One of the latest additions to commercial systems for CTC detection and analysis is Parsortix^®^ PC1, manufactured by ANGLE plc. It is based on a microfluidic chamber and is based on size and deformability of CTCs [98]. The captured cells remain intact for the subsequent separate downstream analysis on the cells, such as cell morphology, DNA, RNA, and protein analysis. The enrichment process is based on the following characteristics of CTC and CTC clusters: unique size and lack of deformability. The system is approved by the FDA for enriching the cells from breast cancer patients [99]. The platform was used to enrich CTCs in many studies that needed a comprehensive liquid biopsy approach. The results of sequencing large‑scale transitions (a type of chromosomal instability) from CTCs from triple‑negative breast cancer patients collected using Parsortix^®^ PC1 showed that it has the potential to reveal more about how dynamic these genomic changes are over time. This could have consequences for tracking triple negative breast cancer (TNBC) advancement through repeated evaluations [100]. One of these works investigated resistance mechanisms of non-small cell lung cancer (NSCLC) to osimertinib, a second-line treatment [101]. The authors used CTCs because they can capture the dynamic molecular changes associated with drug resistance, offering a more complete picture than cell-free DNA (cfDNA) alone. The study included 30 NSCLC patients whose samples were collected both before starting osimertinib and at disease progression. The results revealed several potential resistance mechanisms, some of which were present in CTCs while others were present in cfDNA. These findings suggest that a combined cfDNA and CTC analysis provides complementary information and can identify targets for subsequent treatment strategies with drugs in NSCLC patients progressing on osimertinib. Another study investigated the clinical utility of CTCs in both localized and metastatic prostate cancer [102]. The authors used CTCs because they are a known prognostic marker in metastatic prostate cancer, and they hypothesized that they could also provide valuable information in earlier stages of the disease. After enriching CTCs in the peripheral blood of patients with early intermediate and high-risk prostate cancer and patients with metastatic prostate cancer, two CTC-related markers [PSA and prostate specific membrane antigen (PSMA)] were quantified using qPCR and RNA in situ hybridization (ISH). They concluded that CTC analysis using this marker panel can effectively detect prostate cancer cells, even in localized disease, and that RNA ISH confirms marker expression at the single-cell level. Furthermore, the PSMA marker could be used diagnostically to identify patients who might benefit from PSMA-directed PET-CT scans or PSMA-targeted therapies. The Parsortix^®^ PC1 System was also utilized for capturing CTCs in MBC patients and healthy volunteers [103]. Harvested cells were analyzed using both IF to detect epithelial markers and Wright-Giemsa (WG) staining for morphological assessment. A significant portion of CTCs in MBC patients did not express EpCAM, and CTC clusters were found in 56% of CTC-positive patients. The study concluded that the system can effectively capture CTCs from a greater proportion of MBC patients than healthy volunteers, highlighting its potential for clinical use. The detection of epithelial cells in some people from the control group, while previously observed, remains of unclear clinical significance. The finding that many CTCs did not express EpCAM underscores the limitations of relying solely on EpCAM-based CTC detection methods. Another study compared seven different CTC enrichment methods across five technologies to determine the optimal approach for lung cancer [104]. The study used healthy donor blood (5 mL) spiked with fluorescently labeled lung cancer cell lines (H1975, A549, and H1299 with varying EpCAM expression). The CellMag™ (EpCAM-dependent) had the highest recovery rate (70%) with H1975 cells, but its recovery significantly decreased with A549 (35%) and H1299 (1%) cells, demonstrating EpCAM-dependence. The Parsortix^®^ PR1 (size- and deformability-based) in-cassette staining showed consistent recovery rates across all three cell lines: H1975 (49%), A549 (47%), and H1299 (52%), indicating cell phenotype independence. The Parsortix^®^ PR1 was also shown to isolate heterogeneous single CTCs and cell clusters from patient samples. The study concluded that in-cassette staining method of this platform is optimal for CTC enrichment in lung cancer due to its consistent recovery rates across different cell phenotypes and its ability to capture both single cells and cell clusters, though further optimization and validation are needed.

### Comparing CTC-detecting platforms

There are numerous studies that compared the performance of these systems side-by-side. However, one of the main challenges in comparing these technologies remains the lack of a unified definition of CTCs [86]. According to the mode of use, these systems can be classified into those used in vivo or ex vivo (in vitro). Two of the systems use EpCAM as a ligand to capture the target cells, while Parsortix^®^ is an epitope-independent technology. In terms of approval and clearance by the agencies protecting the public health, these platforms also differ in their approval geographically and the level of validity: Parsortix^®^ is an FDA-cleared device, while CellSearch™ was approved by the FDA; CellCollector™ was approved by the National Medical Products Administration (NMPA) (formerly the China FDA) and is CE certified. One study comparing the performance of CellSearch™ and CellCollector™ in a prospective and investigator-blinded study concluded that CellSearch™ did not outperform the latter in terms of yield and sensitivity in colorectal cancer [105]. Total number of CTC and the frequency as detected by these two methods were not significantly different in both metastatic and non-metastatic colorectal cancer patients, despite the suggested increase of processed blood volume by the CellCollector™. An additional in silico analysis performed by the authors revealed that during in vivo use, guidewire-based platform processes a much lower volume of blood (0.33–18 mL per 30 minutes) than was previously reported. Other reasons for inferior results by CellCollector™ were attributed to the method of positioning the functionalized guidewire, the differences among individuals related to their blood circulation and CTC counts, as well as the somewhat subjectivity in the interpretation of the results. Getting such results, which contradicted a previous work by Theil et al. [106], was attributed to the use of an in vitro setup for testing the device instead of inserting it into patients’ arms, which is understandable. However, the same authors have later published at least two works, but on different types of cancer, where they actually have administered the Gilupi device into patients’ veins and got better capture of CTCs than CellSearch™. The results of the studies using prostate cancer have demonstrated that isolation efficiency using guidewire-based technology was higher than that of the FDA-approved method (65.7% vs. 44.4%) [107]. In another study using blood from castration-resistant prostate cancer, it was shown that CTC detection rates for CellCollector™ were higher [108]. An earlier study found that the guidewire device had a higher number of captured CTC from lung cancer patients than CellSearch™ (58% vs. 27%) [97]. However, some of the published results that show superior performance of CellCollector™ over its FDA-cleared competitor were published only as conference papers. Thus, CellCollector™ has been compared with FDA-approved CellSearch™ and showed higher sensitivity for breast cancer: 74% compared to 12%, and high precision (linear regression, r^2^ = 0.96) [96].

After its recent arrival, a plenty of studies compared the performance of the FDA-cleared Parsortix^®^ system to the one approved by the FDA for CTC analysis. An earlier study reported no significant difference between these platforms in overall cell capture [109]. CellSearch™ captured more CTCs, which are considered traditional CTCs (cytokeratin-positive and CD45-negative cells), than Parsortix^®^ when esophageal cells were spiked in the blood obtained from healthy donors and patients with esophageal adenocarcinoma [110]. However, in many other studies, CellSearch™ showed inferior results when detecting CTCs. Thus, an epitope-independent method system was able to capture significantly more EpCAM-positive cells when the performance was compared to CellSearch™ from prostate cancer patients, although the number of tested samples was limited (ten) [111]. Additionally, a Parsortix^®^ was able to capture CTC clusters. The main strength of a Parsortix^®^ is that its performance is not limited to capturing EMT-dependent CTC but can capture more mesenchymal CTCs, making it an EMT-independent technology [112]. While almost all of the abovementioned studies considered these technologies as “enemies”, some works aim to establish a workflow that has two of the discussed technologies with a modern single-cell analysis. Thus, a work by Lampignano et al. [113] offers an interesting approach of combining CellSearch™ and Parsortix^®^ in an innovative workflow with an additional analysis of mutational status of the captured cells by Sanger sequencing with an ultimate goal of improving breast cancer diagnosis. The main strengths and weaknesses of the systems, as published by the studies, are shown in Table 4.

**Table 4:** Some of the currently available commercial systems for CTC detection and analysis, which were used with clinical samples

**System name**	**Description**	**Use**	**Enumeration/downstream analysis (DA)**	**Certification**	**Some of the reported strengths**	**Some of the reported weaknesses**	**Ref.**
CellCollector™	Functionalized guidewire	In vivo	No enumeration DA can be performed after.	Certified by CE and the NMPA	A large amount of blood is processed. Well-tolerated (no adverse events)	Uncontrollable luminal positioning Difference in blood circulation Delicate/subjective result interpretation Detects only EpCAM^+^ cells	[105]
CellSearch™	Immunomagnetic capture and fluorescence imaging technology	Ex vivo	Limited enumeration Fluorescence imaging technology DA not included (cell images)	FDA approval	A large number of studies Gives quantitative results Standard method	Result interpretation can be subjective. Detects only EpCAM^+^ cells	[87, 114]
Parsortix^®^ PC1	Microfluidic cell-capturing cassette	Ex vivo	DA not included DA can be performed after.	FDA clearance	Label-independent High capture efficiency Viable cells are captured. Cell clusters can be caught.	Need some optimization for optimal performance No enumeration	[98, 104, 111]

CTC: circulating tumor cell; FDA: Federal Drug Administration; NMPA: National Medical Products Administration

## Conclusion and future perspective

Detection of CTCs as part of liquid biopsy is a promising innovative technique that could enable accurate tumor diagnosis and personalized treatment, since CTCs hold promise as a diagnostic, prognostic, and predictive biomarker. Although the first definition of CTCs dates back to 1869, when Ashworth identified these cells, their rarity, heterogeneity, and complexity of the blood pose significant challenges for their isolation and detection. Plenty of ongoing research aims to overcome the inherent challenges associated with these important cells. The employed strategies include improving the sensitivity of the sensors by using innovative designs and materials and improving surface functionality. Other sets of devices aimed at building sensors that do not use fluorescent tags or which provide real-time measurements, paving the way for quicker assays and continuous monitoring of the biomarker.

However, the CTC status of the target cells in these studies was not always confirmed, or only one of the CTC characteristics (EMT-based biomarker or size) was used as a criterion. However, the expression of epithelial markers like EpCAM may be reduced [115, 116]. Therefore, there is a need to find more specific markers and/or use a combination of CTC-associated biomarkers or other characteristics to isolate CTC from liquid biopsy.

The widespread applications of commercially available platforms for detecting these cells in clinical settings have been hindered mainly by the cost of these instruments or existing limitations in the performance of the devices. Also, for the creation of effective and reasonably priced clinical platforms for tracking the growth and spread of tumors by detecting CTCs and performing comprehensive analysis of these cells, it is important to have quality control, standardized CTC detection and characterization methodologies. Difficulties in standardizing the observation of cells captured by such in vivo platforms as CellCollector™ could be overcome by using platforms of the same size but having sensing capabilities, such as optical fiber sensors. Additionally, for sensing technologies to successfully transition from laboratory to clinical practice, partnerships between scientists, regulatory agencies, and biosensor engineers are required.

Table 5 provides a comparative overview of different techniques used for CTC detection, including chip-based systems, electrochemical biosensors, and optical fiber biosensors. Each method is summarized based on its key strengths, limitations, and practical applications in cancer diagnostics and treatment monitoring. While chip-based methods offer high sensitivity, isolation of viable cells, and integration capabilities, they are often costly and complex. Electrochemical and optical fiber biosensors provide promising alternatives with specific advantages, though they also face challenges such as signal interference and limited sensitivity at low CTC counts.

**Table 5:** Summary of various techniques for CTC detection, including their strengths, limitations, and practical applications in cancer diagnostics and monitoring

**Technique for CTC detection**	**Advantages**	**Disadvantages**	**Practical applications**
Chip-based	Allows integration of enrichment, detection, and on-chip culture Enables isolation of viable cells Combining biophysical and biochemical properties Closer to the point-of-care device	Complex design Not in situ Requires precise fluid control and optimization May be costly	Used to detect CTCs in various cancers (gastric, colorectal, breast, lung) Tested for diagnostic and monitoring purposes
Electrochemical biosensors	High sensitivity Real-time, label-free detection Compatible with microfluidic integration	Electromagnetic interference Limited multiplexing Complex fabrication	Used mainly with MCF-7, A549, HeLa cells Tested in PBS, human blood, and clinical samples Applied in prostate, cervical, and liver cancer studies
Optical fiber biosensors	High sensitivity Label-free Real-time Biocompatible material Miniature	Fragile Limited practical use at low CTC counts Costly (TFBG)	Used to detect breast cancer cells (e.g., BT549, MDA-MB-231) Applied to whole blood and serum

CTC: circulating tumor cell

**Figure 2:**
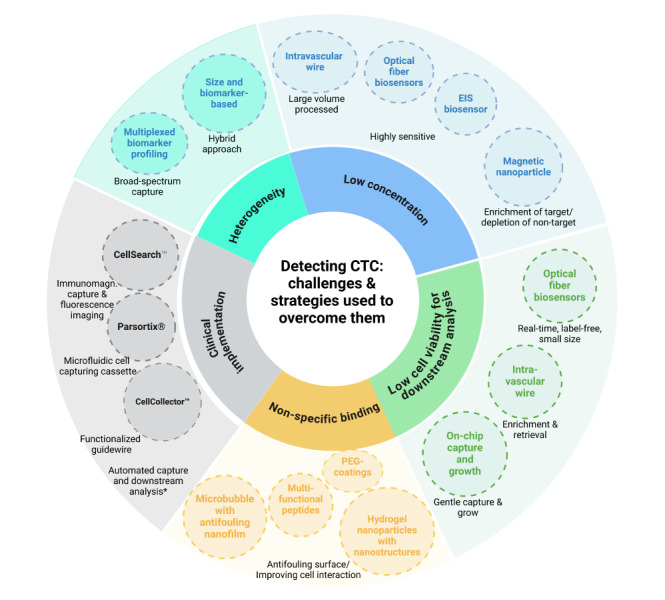
**Overview of the key challenges in CTC detection and the corresponding strategies used to overcome them.** Inner circle: challenges; outer circle: some strategies with real examples from the studies (in dashed circles). Created in BioRender. Bekmurzayeva, A. (2025) https://BioRender.com/wtrmsdx. CTC: circulating tumor cell; EIS: electrochemical impedance spectroscopy; * systems of the future

Figure 2 illustrates the key challenges in CTC detection and the specific strategies developed to address them. These challenges include the low abundance of CTCs in blood, heterogeneity among CTC populations, limited cell viability for downstream applications, non-specific binding, and barriers to clinical implementation. To overcome these obstacles, various methods have been developed, each targeting a specific issue. For example, intravascular wire systems enhance cell viability by enabling gentle enrichment and retrieval of the cells after processing a large amount of blood to capture rare cells. On the other hand, high sensitivity, label-free nature, and real-time detection capability of optical fiber biosensors address the issues of extreme rarity and cell viability of CTCs.

In the future, using platforms already established in a clinical setting to capture CTC might improve early diagnosis, prognosis, and therapy of cancer patients. Moreover, further molecular characterization of the captured cells could bring us a step closer to personalized medicine. Integrating the platforms for continuous CTC detection into clinical practice could give us more insight into the dynamic changes of these cells during the treatment regimens, which then could aid doctors to make informed changes to the treatment, creating a positive feedback loop. Such an approach could be crucial for directing further steps, especially in the case of weakened patients. It is also possible that the devices of the future will be directly integrated with the proteomics, genomics, and other -omics approaches, which could give real-time, more comprehensive information on the status of the disease.

## Abbreviations

cfDNA: cell-free DNA
CGO: carboxylated graphene oxide
CTCs: circulating tumor cells
EIS: electrochemical impedance spectroscopy
EMT: epithelial-to-mesenchymal transition
EpCAM: epithelial cell adhesion molecule
FDA: Federal Drug Administration
FISH: fluorescent in situ hybridization
GEs: gold electrodes
IF: immunofluorescence
IHC: immunohistochemistry
ISH: in situ hybridization
LAPS: light-addressable potentiometric sensor
MBC: metastatic breast cancer
NSCLC: non-small cell lung cancer
PET: polyethylene terephthalate
PSA: prostate specific antigen
PSMA: prostate specific membrane antigen
RCA: rolling circle amplification
SERS: surface-enhanced Raman scattering
TDSs: thiolated DNA strands

## Declarations

### Copyright

© The Author(s) 2025.
